# Potential Biomarkers of Fat Loss as a Feature of Cancer Cachexia

**DOI:** 10.1155/2015/820934

**Published:** 2015-10-05

**Authors:** Maryam Ebadi, Vera C. Mazurak

**Affiliations:** Division of Human Nutrition, Department of Agricultural, Food and Nutritional Science, University of Alberta, 4-002 Li Ka Shing Centre for Health Research Innovation, Edmonton, AB, Canada T6G 2E1

## Abstract

Fat loss is associated with shorter survival and reduced quality of life in cancer patients. Effective intervention for fat loss in cachexia requires identification of the condition using prognostic biomarkers for early detection and prevention of further depletion. No biomarkers of fat mass alterations have been defined for application to the neoplastic state. Several inflammatory cytokines have been implicated in mediating fat loss associated with cachexia; however, plasma levels may not relate to adipose atrophy. Zinc-*α*2-glycoprotein may be a local catabolic mediator within adipose tissue rather than serving as a plasma biomarker of fat loss. Plasma glycerol and leptin associate with adipose tissue atrophy and mass, respectively; however, no study has evaluated their potential as a prognostic biomarker of cachexia-associated fat loss. This review confirms the need for further studies to identify valid prognostic biomarkers to identify loss of fat based on changes in plasma levels of biomarkers.

## 1. Introduction

Cancer cachexia is associated with increased mortality and morbidity in cancer patients [1]. By international consensus, cancer cachexia is proposed to be “a multifactorial syndrome defined by an ongoing loss of skeletal muscle mass with or without loss of fat mass that cannot be fully reversed by conventional nutritional support and leads to progressive functional impairment” [2]. A recent review [3] reported elevated lipolysis to be the major reason for fat loss in cancer cachexia [4, 5] although the underlying mechanisms are undefined. As cancer progresses, the majority of patients experience loss of fat. Fat loss precedes muscle loss, associates with shorter survival [6, 7], and is variable with respect to timing and intensity in various cancer populations [3]. Therefore, identification and validation of markers of fat loss are crucial not only for a better understanding of mechanisms, but also to identify fat losing cancer patients who will subsequently develop cachexia. Effective management of cancer cachexia is restricted to early identification of the syndrome; therefore, biomarkers are vital for development of appropriate therapeutic interventions to achieve better outcomes for individual cancer patients.

Adipose tissue (AT) is an active secretory organ, composed mainly of adipocytes and nonadipocyte cells such as inflammatory cells, immune cells, preadipocytes, and fibroblasts [8]. Adipokines are proteins synthesized and secreted from adipocytes which act both locally and distally, contributing to whole body lipid metabolism [9, 10]. In pathophysiological conditions like cancer, macrophage infiltration into AT increases [11, 12], leading to alterations in adipokine production affecting adipose tissue mass and function. Local adipokines produced by AT, circulating cytokines, and lipid mobilizing factors are collectively involved in adipose atrophy in cancer cachexia [13, 14]. Considering adipose tissue as a metabolically active organ as well as the relationship between fat loss and shorter survival in cancer, early identification of fat losing patients may increase the opportunity for therapeutic management of cachexia.

Biomarkers can be applied to represent tissue alterations under both physiological and pathological conditions [15]. A biomarker is “a biological molecule found in blood, other body fluids, or tissues that is a sign of a normal or abnormal process or of a condition or disease.” [16]. Biomarkers indicate normal biologic processes, pathogenic processes, or pharmacological responses to a treatment [15]. Biomarkers in the oncology setting, identified using high-throughput sequencing, gene expression arrays, and mass spectroscopy [17], are classified into prognostic, predictive, and pharmacodynamic categories [18–20]. Prognostic biomarkers provide information about likely outcome of a disease, regardless of treatment, whilst predictive biomarkers assess the effect of a particular treatment. Pharmacodynamic biomarkers assess drug treatment effects on a tumour [18–20]. Ideal biomarkers are easily accessible, available, specific and sensitive, noninvasive, inexpensive, consistent, safe, and easy quantifiable in a biological fluid or clinical sample. Biomarkers are consistent across genders and ethnic groups. Levels of the biomarker should not overlap between controls and patients while significantly relating to the outcome of interest using appropriate statistical analysis [18].

While it seems important to identify a prognostic biomarker of cancer cachexia-associated fat loss, no ideal clinical biomarker has been defined yet, which demonstrates a need to identify and subsequently validate potential biomarkers in independent studies. Studies focusing on adipose tissue have identified leptin, free fatty acids (FFAs), and glycerol in plasma as indicators of fat alterations in health and diseases. On the other hand, adipokines including inflammatory cytokines such as interleukin-6 (IL-6) and tumour necrosis factor-*α* (TNF-*α*) [21] as well as Zinc-*α*2-glycoprotein (ZAG) [22] have also been associated with weight and fat loss in cancer. Therefore, circulating levels of these factors may represent new noninvasive prognostic biomarker of adipose atrophy and targets in the detection and management of fat loss in cancer.

One of the major obstacles to identify reliable biomarkers of fat loss in cancer cachexia is variation between studies in how fat loss is assessed. Body mass index (BMI) is frequently used as a clinically accessible measure of human body composition. However, as BMI does not distinguish between fat and fat-free mass, its utility in the settings of fat loss in cancer cachexia is limited [23]. Various methods including bioelectrical impedance analysis (BIA), dual-energy X-ray absorptiometry (DEXA), magnetic resonance imaging (MRI), and computed tomography (CT) scan analysis [24] have been applied to assess body composition in cancer population. CT image analysis, as the gold standard for body composition assessment in cancer patients, has an ability to discriminate and precisely quantify different adipose tissue depots. Many patients have repeated scans over the cancer trajectory enabling assessments in the same individual over time. Application of body composition assessment in the cancer setting has focussed primarily on lean body mass. The studies that do exist reveal loss of adipose tissue as cancer progress [25, 26]. However, further studies are required to establish the timeline and pattern of fat mass alterations in different adipose tissue depots during cancer progression [3]. Moreover, the majority of studies assessing fat mass focus on gastrointestinal cancer patients; there remains a gap in knowledge related to other malignant tumours. Finally, timing of CT scans differs between patients and scans may not be available over a specific time points demonstrating the need for other important prognostic biomarkers of fat loss. Overall, gaps remain related to the association between fat mass alterations assessed by CT scans and circulating markers of fat loss. This article reviews current knowledge around potential prognostic biomarkers of fat loss in cancer which may identify fat-losing cancer patients who would benefit from early therapeutic interventions to improve outcome of cancer patients. Possibilities and potential to apply these markers as prognostic biomarkers of fat loss will be discussed.

## 2. Inflammatory Cytokines 

Serum levels of cytokines associate with clinical features of cancer cachexia such as weight loss; however, no study has specifically assessed the association between serum cytokines and the extent of fat loss in cancer patients. Inflammatory cytokines, such as IL-6 and TNF-*α*, are produced by tumours and by nonfat cells residing in AT [21] in addition to adipocytes. Plasma levels of inflammatory cytokines are elevated in cachexia [27] and are thought to promote adipose atrophy in animal and human models of cachexia [13]. Pathways of adipose tissue metabolism evoked by IL-6 and TNF-*α* include inhibition of lipoprotein lipase mRNA expression and activity which prevents fat cells from taking up fatty acids from lipoproteins [28, 29]. These cytokines stimulate hormone sensitive lipase (HSL) and adipose triglyceride lipase (ATGL) activity [30, 31], leading to elevated lipolysis. TNF-*α* has been reported to prevent preadipocyte differentiation [32] and inhibit expression of lipogenic transcription factors [33]. Collectively, these alterations would result in fat loss.

Serum TNF-*α* levels negatively correlate with body weight and BMI in pancreatic cancer patients [34]. Tumour presence has been associated with elevated serum IL-6 and TNF-*α* in mice bearing the Lewis lung carcinoma or B16 melanoma cells compared to controls [35]. In humans, data regarding the role of TNF-*α* in cancer-associated wasting are controversial. Measuring TNF-*α* in plasma is challenging due to short half-life and transient nature. Further, the sensitivity of assays used to measure plasma TNF-*α* is variable, making comparisons between studies limited [36]. On the other hand, TNF-R1 and TNF-R2 (soluble TNF-*α* membrane receptors) have been applied as serum markers of TNF-*α* activity due to their longer half-life and greater stability [37].

A comprehensive review of clinical factors associated with cachexia [38] showed little evidence for the association between serum TNF-*α* and weight loss in cancer, while several studies report an association of plasma IL-6 but not TNF-*α* with cachexia-associated wasting rather than cancer per se. Serum IL-6 levels were higher in fat losing gastrointestinal cachectic cancer patients compared to weight stable and noncancer controls. However, no changes in mRNA expression or secretion of IL-6 and TNF-*α* from SAT were observed [4]. This finding was confirmed in another study showing that circulating IL-6 levels were higher in weight losing non-small-cell lung carcinoma patients compared to weight stable cancer patients [39].

Adipose atrophy has been associated with elevated IL-6 signalling in a preclinical model of cancer cachexia [40]. In patients with gastrointestinal cancer, plasma IL-6 levels significantly correlated with the presence of tumour and increased with each progressive stage of cancer [41]. IL-6 has been reported to be involved in early stages of cachexia [42, 43] and a study conducted in patients with mixed tumor types showed IL-6 levels gradually increased during early stages of cachexia followed by rapid increase prior to death [44]. In contrast, a study in 61 patients with advanced cancer showed no correlation between IL-6, TNF-*α*, and weight loss [45]. Although circulating IL-6 levels were higher in cachectic mice compared to controls [46], IL-6 receptors deficient (IL-6-R-KO) mice were partially protected so other cytokines may involve in cachexia-associated wasting. Moreover, a study published in 2012 reported that other cytokines, such as IL-1*β* but not IL-6, may be better indicator of cachexia features such as weight loss and body composition alterations [43].

Collectively, evidence would suggest that inflammatory cytokines are involved in AT depletion in cancer [13, 36, 42]; however, plasma concentrations may represent the presence of a tumour rather than cachexia-associated adipose atrophy per se [41]. Future studies are required to assess changes in adipose tissue depots, both visceral adipose tissue (VAT) and subcutaneous adipose tissue (SAT) over the disease trajectory using validated body composition assessment tools and correlating those to changes in circulating cytokines. Given that there could be various sources of cytokines contributing to plasma levels, the transient nature of cytokines, as well as the cost associated with cytokine measures, the application of plasma measures of cytokines as biomarkers of adipose tissue atrophy in the clinical study is likely limited. Moreover, the ability of cytokines to evoke cancer cachexia depends on tumour type and the complex response within a network of mediators, rather than a single cytokine [47, 48]. Major gaps remain regarding the association between plasma cytokine levels and fat loss, clinical ranges of abnormal measures, and method sensitivity.

## 3. Leptin

Leptin is an adipokine, produced mainly by adipocytes [49]. Leptin regulates body weight by activating the anorexigenic neuropeptides and inhibiting the orexigenic neurons such neuropeptide Y (NPY) [50, 51]. Besides body weight and fat mass regulation, leptin is involved in immune function and inflammation [52]. Normally, a lower plasma concentration of leptin is associated with higher NPY secretion; however, NPY pathways have been reported to be dysfunctional in anorectic tumour-bearing rats [53]. Many factors influence leptin synthesis and secretion in adipocytes such as insulin, TNF-*α*, glucocorticoids, reproductive hormones, and prostaglandins [54, 55]. In humans, the main factor influencing plasma leptin concentration is adipose tissue mass.

A higher concentration of serum leptin in obese individuals is associated with increased fat mass and cell size [10]. Serum leptin is considered to be an accurate, reliable, and highly correlated measure of total body fat [56]. In healthy subjects [57], elderly adults [58], and obesity [52], plasma leptin levels have been shown to be a precise measure of adiposity. A relationship between low fat mass and low plasma leptin levels has also been reported in cancer patients [59–67]. Advanced gastrointestinal and lung cancer patients experiencing cachexia-associated adipose atrophy exhibited hypoleptinemia [67–69]. On the other hand, breast and gynaecological cancer patients exhibited elevated plasma leptin levels that related to the elevated levels of sex hormones and receptors, rather than cachexia per se [70].

Circulating leptin concentrations have been used as an indicator of fat mass; however further studies are required to examine changes in leptin concentrations that occur throughout the disease trajectory and relative to body fat mass alterations. Longitudinal studies that employ a precise measure of body fat would enable determination of whether changes in plasma levels of leptin change proportional to fat mass alterations. An added level of complexity is that leptin is secreted by both VAT and SAT, with SAT contributing the majority of leptin to plasma due to its larger contribution to overall body mass [65]. Therefore, measures of changes in leptin concentrations over time do not currently represent the type of fat being lost or gained.

Comparison between studies is limited by different assay sensitivities and how leptin values are reported as total, free, or bound leptin. Further, factors such as the type of cancer, BMI, and sex and age influence serum leptin concentration, as reported in adolescents [71], also need to be considered in study interpretation. Low leptin concentrations could be considered a result, not a cause of cachexia, which significantly relates to adipose atrophy and low fat mass in cachexia.

## 4. Plasma Glycerol

Studies indicate elevated lipolysis to be the main cause of fat loss in cancer [4, 5, 72–75]. During AT lipolysis, FFAs and glycerol molecules are produced by the action of lipolytic enzymes such as ATGL and HSL, which hydrolyze stored triglyceride [30]. Adipose atrophy has been associated with elevated activity of ATGL and HSL in human and animal models of cancer [35, 40, 46]. Elevated lipolysis produces higher plasma glycerol in cachectic cancer patients compared to healthy subjects [74] or weight-stable controls [5]. Lipolytic activity was assessed in 13 cachectic and 14 weight-stable cancer patients by assessing circulating glycerol levels (*μ*mol/L/Kg body fat) as an indicator of in vivo lipolysis. Cachexia was defined as >5% weight loss over 3 months or >10% within the previous 6 months. Body fat mass, assessed using BIA, showed lower body fat (% and kg) in the cachectic group compared to weight stable patients. Elevated levels of plasma glycerol, FFAs, and higher expression of genes involved in energy turnover pathways and oxidative phosphorylation revealed increased lipid mobilization from subcutaneous adipose tissue in the cachectic group [72]. These results support those of Agustsson et al. [76] who showed plasma glycerol and FFAs to be higher in newly diagnosed gastrointestinal cancer patients with cachexia who had low body fat mass (kg), assessed using CT images, compared to the weight-stable group. Higher plasma glycerol and FFAs in the cachectic group positively correlated with percent weight loss and negatively correlated with visceral adipose tissue area [76].

Plasma glycerol values in cancer cachectic patients have been reported as *μ*mol/L [77] or *μ*mol/L/Kg body fat [4, 5, 72, 76]. Interestingly, studies focusing on lipolytic activity in cancer cachexia report a narrow range of plasma glycerol for cachectic patients between studies [4, 5, 72, 76], strengthening its use a potential biomarker. Plasma glycerol has been reported as 6.2 ± 2.7 [5], 6.9 ± 1.3 [76], 7.0 ± 4.3 [72], and 9.8 ± 2 [4] (*μ*mol/L/Kg body fat) in cachectic patients compared to weight stable cancer patients reported at 3.1 ± 0.7 [5], 3.9 ± 0.6 [76], 3.4 ± 1.6 [72], and 3.3 ± 0.3 [4] (*μ*mol/L/Kg body fat). Postabsorptive whole body lipolytic rate, assessed by glycerol infusion technique, revealed basal levels of plasma glycerol to be higher in a cancer group compared to controls. While lipolytic rates were similar, glycerol clearance rate varied between the two groups and contributed to higher glycerol levels. Although preillness weight loss ranged from 0 to 20% in cancer patients, the same results were obtained when data was corrected for body weight [78].

Despite the use of plasma glycerol as an index of whole-body lipolysis, caution should be exercised when considering the results of these studies. Lipolysis results in the release of fatty acids and glycerol from adipose tissue, with glycerol being a better index of lipolysis as FFAs liberated by lipolysis may be reesterified within adipose tissue [79]. AT has very low glycerol kinase activity [80], and glycerol released by lipolysis enters into the bloodstream. However, lipolytic activity is not specific to adipose tissue and occurs also from intermuscular triglyceride stores and plasma lipoproteins [79]. Glycerol concentration may indicate that lipolysis occurs in SAT as glycerol released from visceral adipose tissue lipolysis enters the liver via the portal vein [81]. Therefore, plasma concentrations of glycerol reflect the balance between glycerol release by lipolysis (predominantly adipose tissue) and clearance of glycerol by liver [79] and should be interpreted with caution.

## 5. Zinc-***α***2-glycoprotein 

ZAG is a protein discovered in human plasma [82] that has been associated with presence of several types of carcinomas such as breast, prostate, and lung [83–85]. Elevated serum ZAG, as a routine and reliable measurement, may apply to early diagnosis of cachectic cancer patients with adipose atrophy [86]. ZAG has been considered as an adipokine involved in lipid metabolism in adipose tissue [87, 88]. Both in vivo and in vitro studies have shown that increased ZAG expression in adipose tissue is associated with increased lipolysis and subsequent fat and weight loss [89, 90]. The exact mechanism by which ZAG participates in fat loss in cancer is not known. ZAG may induce lipolysis through activation of *β*-adrenoreceptors [89, 91] and elevated HSL activity [92, 93]. Although the mechanism behind ZAG regulation in AT is still unknown, glucocorticoids have been suggested to stimulate ZAG expression in AT [94]. Increased plasma cortisol levels in cachectic tumor bearing mice [93] and in cancer patients [95] have been associated with higher AT ZAG expression and elevated lipolysis. This implies that, in cachexia, glucocorticoids may induce lipolytic activity through an increase in ZAG expression [94, 96].

There is discrepancy in the association between circulating ZAG levels and weight or fat loss in various conditions. Data on serum ZAG levels in obesity are inconsistent, being reported as either increased [97] or decreased [98] which positively and negatively correlated, respectively, with BMI. Elevated serum ZAG levels have been observed in chronic heart failure and haemodialysis patients suggesting ZAG to be a marker of fat catabolism [22]. In contrast, two studies in cancer patients [77, 92] demonstrated that plasma ZAG levels may not be a good biomarker of cachexia-associated features such as weight and fat loss. Twenty-five GI cancer patients underwent curative abdominal surgery and were categorized as cachectic or weight stable. Cachexia was defined as unintentional weight loss of more than 5% during the previous 6 months. mRNA and protein levels of ZAG in subcutaneous adipose tissue were higher in cachectic cancer patients compared to weight-stable cancer patients which significantly correlated with fasting serum glycerol levels and weight loss. In this study, however, there was no significant difference in circulating ZAG levels between cachectic and weight stable cancer patients. Production of ZAG by tumours and nonadipose tissue, such as the liver, may also affect ZAG plasma levels [92]. This result is consistent with Rydén et al. [77] who report that ZAG is a locally produced factor, promoting AT lipolysis, but not secreted predominately to circulation [77]. Therefore, circulating levels of ZAG are not likely to relate to fat loss in cancer cachectic patients but instead may mediate local lipid mobilising action in adipose tissue.

## 6. Conclusion

Patients with advanced cancer frequently suffer weight and fat loss. Accelerated loss of adipose tissue is associated with shorter survival, reduced quality of life, and decreased muscle mass during cancer progression [6]. Due to the role of adipose tissue in mediating human metabolism, identification of prognostic biomarkers of fat loss in cancer may help to identify fat losing cancer patients for early therapeutic interventions, improved survival, and prevention of muscle atrophy in cancer patients.

No studies in cancer have identified a prognostic biomarker of fat mass alterations nor have the sensitivity, specificity, and reproducibility of potential indicators been assessed in the neoplastic state. Inconsistency in the literature may be due to varying sensitivity of assays used to measure plasma levels of mediators, heterogeneity of patient populations and treatment, and various body composition assessment methods. Inflammatory cytokines appear to be mediators of cachexia-associated features such as fat loss [13, 36, 42]; however, they do not fulfill several components of biomarker criteria. Relationship between circulating cytokines and degree of fat loss in cancer has not been assessed. ZAG in plasma has been suggested to indicate the presence of some type of tumours, and in AT, ZAG can act locally to modulate lipolysis. Literature regarding the potential of plasma ZAG to be a biomarker of fat loss during the development of cancer cachexia is inconsistent. Enhanced adipose tissue ZAG expression in cancer cachexia suggests that ZAG could be a local catabolic mediator within the tissue rather than being a biomarker of fat loss [77]. Therefore, the ability of ZAG to be applied as a marker of lipid utilization in cachexia syndrome and to indirectly represent fat loss is limited.

Plasma glycerol and leptin may have potential to be considered as biomarkers of lipolysis and fat mass, respectively; however, no study has defined a confirmed range and optimal cut-off points for these markers. It is not clear whether a single biomarker or combination may have the most prognostic value, as no study has assessed various combinations in a cancer population. Measuring changes in fat mass over time concurrent with circulating levels of biomarkers of fat mass would provide valuable information about application of proposed fat loss biomarkers throughout the disease trajectory. These studies would help establish valid criteria to identify loss of whole body fat mass based on changes in plasma levels of these specific biomarkers.

Alterations in fat mass and composition between visceral and subcutaneous depots are divergent and vary over the cancer trajectory. The proportional reduction of each fat depot may be a consideration when establishing biomarkers. For example, it remains to be determined whether decreased leptin levels indicate the loss of visceral or subcutaneous adipose tissue in cancer. Future studies should consider the metabolic differences between these depots in determining specific biomarkers.

Although many of the proposed biomarkers are economical, easy, and quick to quantify in plasma, further steps such as comparison of plasma levels in healthy, weight stable, and weight losing cancer patients as well as their correlation with various degrees of fat loss assessed by CT images should be considered in determining capacity for application of a prognostic biomarker of fat loss in cancer. Proper study design, combined with extensive testing, and quantitative measurement of large numbers of proteins in body fluids using advanced techniques [99] as well as statistical validation of prognostic biomarkers [100] are important factors in identification of fat loss biomarkers. This review confirms the need for further studies to (1) assess how alterations in fat mass is reflected in measurable biomarkers, (2) minimize variations that may confound establishment of a biomarker, and (3) increase specificity and sensitivity of methods to detect biomarkers in samples at minimum levels or in repeated measures.
